# “In God We Trust”: An Exploratory Study of the Associations Between Religiosity and the Caregiving Experiences of Parents of Children with Rare Diseases in Poland

**DOI:** 10.1007/s10943-024-02095-4

**Published:** 2024-08-05

**Authors:** Jan Domaradzki, Dariusz Walkowiak

**Affiliations:** 1 Department of Social Sciences and Humanities, Poznan University of Medical Sciences, Rokietnicka 7, St, 60-806 Poznań, Poland; 2 Department of Organization and Management in Health Care, Poznan University of Medical Sciences, Poznań, Poland

**Keywords:** Caregiving burden, Coping, Children, Experiences, Family caregivers, Rare diseases, Religiosity

## Abstract

Most children with a rare disease are cared for by their family members but parenting such a child is extremely demanding due to the complexity and severity of symptoms, with serious physical, emotional, social, and financial consequences for caregivers. Although religion may serve as a positive coping strategy, little is known about its role in helping caregivers manage the stress related to the burden of caregiving in Poland. Therefore, we surveyed 925 Polish family caregivers of children with rare diseases to understand the association between caregivers’ religiosity and their caring experiences. The findings suggest that parents’ religiosity is associated with a more positive caregiving experience, perceived quality of life, and experienced caregiving burden. While religious caregivers reported experiencing less distressing emotions and stressed the encouraging impact of their child’s disease on their life more often, non-religious caregivers experienced role captivity and role overload more frequently. Since religion may serve as a source of strength and a protecting factor against mental health problems and the burden of caregiving, healthcare professionals should be aware of the importance of religious and spiritual care, and caregivers’ religiosity should be considered an integral part of a holistic approach.

**Supplementary Information:**

The online version contains supplementary material available at 10.1007/s10943-024-02095-4.

## Introduction

As in other European countries, a condition is considered rare in Poland when it affects fewer than 1 per 2000 people within the general population (Ministerstwo Zdrowia, 2021). Although rare diseases (RDs) seem to be rare it is estimated that there are over 7,000 distinct conditions (Haendel et al., 2020; Rare-X, 2022). Some, however, report as many as 10,867 RDs (Rare-X, 2022), of which, many are classified as ultra-rare (a prevalence of fewer than 1 per 50,000 persons), hyper-rare (a prevalence of fewer than 1 per 1,000,000,000 persons) (Hennekam, 2011; Harari, 2016; Smith et al., 2022) or as *Syndrome Without A Name* (SWAN), that is a condition so rare that it may never have been seen before (Aldiss et al., 2021; Jewitt et al., 2017). This means that RDs constitute as many as 1/10 of all human diseases (The Lancet Diabetes Endocrinology, 2019), affecting between 6 and 8% of the global population (Auvin et al., 2018). Approximately 80% of all RDs are of genetic origin and 75% affect children. Since 95% of RDs have no approved treatment, it is estimated that 50% of children with a RD die before they reach five years of age, and 35% before the age of one (Marwaha et al., 2022).

Approximately 30 million people in the European Union have a rare disease (European Commission, n.d.) with between 2 and 3 million in Poland alone (Ministerstwo Zdrowia, 2021). However, many other sufferers remain unrecognized and struggle for a diagnosis and treatment (Libura et al., 2016). Consequently, RDs have been recognized as a serious public health issue and the Polish government implemented a *National Plan for Rare Diseases* in 2021. While the plan is still in consultation, its main objectives are to create a Polish Register of Rare Diseases, improve access to modern diagnostic methods using large-scale genomic tests that will guarantee timely and accurate diagnosis, improve access to modern treatment and products, that is, medicines, medical devices and foodstuffs for patients with special nutritional needs, introducing the so-called “Rare Disease Patient Passport” and the creation of a Rare Diseases Information Platform (Ministerstwo Zdrowia, 2021; Kancelaria Prezesa Rady Ministrów, 2024).

Although the National Plan for Rare Diseases follows the EU’s recommendation obliging all Member States to develop therapeutic strategies for RDs Poland has yet to introduce support mechanisms for such patients. Consequently, many RD patients are neglected by the healthcare system and struggle for a timely diagnosis and access to proper treatment (Black, Martineau & Manacorda, 2015; Carmichael et al., 2015; Bauskis et al., 2022; Llubes-Arrià et al., 2022; Teutsch et al., 2023; Witt et al., 2023). Moreover, many healthcare professionals in Poland lack knowledge of RDs (Walkowiak & Domaradzki, 2020, 2021), therefore there is an urgent need to raise healthcare professionals’ awareness of RDs. However, since the National Plan for Rare Diseases focuses primarily on the patients and contains no regulations on social support for their caregivers, they should be educated regarding the situation of caregivers, as most children with RDs in Poland are cared for in their homes and by family members, especially parents, relatives, and friends (Ministerstwo Zdrowia, 2021; Kancelaria Prezesa Rady Ministrów, 2024).

The medical care for children with RDs has improved leading to earlier diagnosis and better treatment, which in turn has resulted in increased life expectancy and decreased risk of premature death (Gorini et al., 2021). However, children with RDs remain overrepresented in pediatric mortality statistics and for many parents, the role of caregiver may last for many years or even decades. Due to the severity and complexity of symptoms which cause numerous physical, mental, emotional, and behavioral problems, caregiving has also become an extremely complex and demanding task that may lead to a caregiving burden (Boettcher et al., 2021; Sandilands et al., 2022; Modrzejewska-Zielonka et al., 2022; von der Lippe et al., 2022; McMullan et al., 2022; Valcárcel-Nazco et al., 2022; Atkins & Padgett, 2024).

The caregiver burden, defined as a “multidimensional response to the negative appraisal and perceived stress resulting from taking care of an ill individual” (Kim et al., 2012), affects caregivers’ physical, psychological, emotional, and functional health and may lead to a deterioration in care and undermine the quality of life of both the caregiver and children with RDs. Thus, it is often argued that caring for a child with RD creates two victims: the child and the caregiver (Bonner et al., 2017). Indeed, disease progression leads to a deterioration of the child’s physical, mental, and cognitive health, so caregiving becomes progressively demanding and stressful with serious physical, psychological, emotional, social, and financial consequences for caregivers (Boettcher et al., 2020; Sandilands et al., 2022; von der Lippe et al., 2022; Mcmullan et al., 2022; Atkins & Padgett, 2024). Furthermore, the caregiver burden includes both objective and subjective dimensions of caring for children with RDs: the former refers to the time and energy spent on the physical aspects of caregiving, while the latter refers to the caregiver’s experience of burden, for example, their perception and emotional reactions to caregiving (Fekete et al., 2017). The caregiver’s perception of the severity of the child’s symptoms and the caregiver burden may at the same time be more important to consider than the actual health problem.

While previous research has focused on the impact of caring for children with RDs on parents’ physical and mental health, their quality of life, and the perceived burden, much less is known about caregivers’ coping resources. Since many children with RDs caregivers lack institutional and professional support, relying on external social support outside the healthcare system (Boettcher et al., 2021), their quality of life is impacted not only by psychosocial strains and their burden but also available coping strategies (Sandilands et al., 2022; Wiedebusch et al., 2008). While many caregivers stress the importance of psychological support, coping strategies, and stress management, some parents of children with RDs prefer to use problem-focused and emotion-focused coping strategies and rarely rely on religious coping strategies (Picci et al., 2015; Sandilands et al., 2022).

Nonetheless, although also religiosity has been recognized as a vital and valuable adaptive resource (Pargament et al., 1998) for caregivers who use it to cope with their loved one’s disease and stressful life circumstances (Durà-Vilà et al., 2010; Grossoehme et al., 2010a, 2012; Nematollahi et al., 2021; Purcell et al., 2015; Vitale, 2016; Wu et al., 2020; Xavier & Esperandio, 2023), still little is known about the role of religiosity as a mediating factor and coping resource that helps parents to manage the stress related to parenting a child with RD in Poland. Meanwhile, multiple studies have demonstrated that religious beliefs and practices, including prayers and church attendance, are positively associated with emotions experienced by the caregivers, and higher level of satisfaction from providing care (Dogan, 2016; Grossoehme et al., 2010a; Pargament et al., 2000; Tabolli et al., 2010; Vitale, 2016; Wu et al., 2020). It was also shown that positive religious coping increases caregivers’ mood, mental and spiritual well-being, but also lowers their level of feeling of burden and enhances caregivers’ quality of life (Calvo et al., 2011; Hebert et al., 2007; Khanjari et al., 2018; Pearce et al., 2016; Yoon et al., 2018). On the other hand, negative religious coping, i.e. spiritual struggle, conflict with religious others, feeling abandoned by or anger at God, or guilt, was associated with more depressive symptoms, increased anxiety, feeling of hopelessness and loneliness, higher level of burden and decreased quality of life among caregivers (Grossoehme et al., 2010b; Daher-Nashif et al., 2020; Malcolm et al., 2021; Nicholas et al., 2017; Chong, Khalid & Abdullah, 2022; Leidl et al., 2023).

At the same time, while religion and spirituality have been recognized as a significant part of holistic care for caregiver of children with RDs, research demonstrated that caregivers’ religious and spiritual needs are often unmet, and not addressed sufficiently by healthcare professionals, which can create additional stress and lower parents’ well-being (Gómez-Zúñiga et al., 2021; Pearce, 2005; Selman et al., 2018).

While over the past few decades, secularization has resulted in the decline of religious practices in Poland and especially after the COVID-19 pandemic, many people do not practice regularly or never go to church, rejecting the Church’s mandates on individual morality (Centrum Badania Opinii Społecznej, 2022a, 2022b), nonetheless, Poland remains one of the most devout countries in Europe, with 71.3% of Poles identifying as Roman Catholics, while only 6.87% declare that they belong to no faith (Statystyczny and [Central Statistical Office], (2023). Since religious faith and practices have been identified as a useful coping resource for caregivers, this study was designed to investigate the association between Polish caregivers’ religiosity and their experiences related to parenting a child with a RD. In particular, it aims to explore the association between parents’ religiosity and (1) caregiving experience and emotions, (2) perception of the caregiving role, (3) perceived quality of life, and (4) perceived caregiving burden.

## Methods

### Study Design

While this study is part of a larger project focusing on the parental challenges related to caring for a child with a RD, it presents data from a self-administered, anonymous, computer-assisted, online survey regarding the association between declared religiosity and the experiences of Polish parents of children with RDs.

### Participants and Setting

The survey was conducted between October 2022 and May 2023 among family caregivers of children with RDs. Since Poland still lacks a registry of persons with RDs and the exact number of pediatric RD patients is unknown, eligible caregivers were recruited with the support of several RD foundations, patients’ associations, and organizations via their web pages and Facebook. The inclusion criteria included being a parent or family member of a child with a RD, providing care for a child aged between 0 and 18 years of age, being able to use electronic devices and participate in an online survey, and providing informed consent.

*Ethical Issues.*

This study complies with the ethical standards of the Declaration of Helsinki (revised in 2000) (Sawicka-Gutaj et al., 2022) and was performed according to the Poznan University of Medical Sciences (PUMS) Bioethics Committee approval (KB – 833/22, 22 October 2022). All caregivers who volunteered to participate received an invitation letter and were informed about the survey's purpose and methods. They were also informed about the voluntary, anonymous, and confidential character of the study, as well as the possibility to contact the research coordinator with any questions or doubts regarding the survey, or for assistance in completing this questionnaire. Participants were also informed about the possibility of quitting the survey at any given time or refusing to reveal information regarding their circumstances without any consequences. Caregivers were also instructed that because some questions relate to sensitive issues that might cause distress, should they experience any emotional pain or stress while recalling past events related to caregiving, they may take the time to collect themselves and decide whether to continue the survey. Finally, participants were informed that all information collected would be used only for scientific purposes. All participants provided written consent.

### Research Tool

Because RDs differ significantly in terms of clinical manifestation, variety and severity of symptoms that affect their physical, cognitive, mental, behavioral, and functional health, the disease course, and prognosis, even between individuals affected by a single disease, parents of children with RDs experience different emotions, needs, and challenges. Consequently, there is no specific tool for the assessment of the caregiver burden of parent caregivers, so an original closed-ended questionnaire was developed after a thorough analysis of the literature (Bonner et al., 2017; Boettcher et al., 2021; Sandilands et al., 2022; Modrzejewska-Zielonka et al., 2022; von der Lippe et al., 2022; Mcmullan et al., 2022; Valcárcel-Nazco et al., 2022). Although the questionnaire was an ad hoc tool, its development followed the guidelines of the European Statistical System (Eurostat, 2005). After the preliminary questionnaire was drafted, we consulted a pediatrician, a public health specialist, and a medical sociologist. It was then pre-tested via an online platform with ten caregivers and re-evaluated, which resulted in the re-phrasing of five items before it was re-evaluated by three additional specialists from the same fields.

The final version of the questionnaire was divided into several parts: the first section asked questions about the demographic characteristics of caregivers and children with RDs; the second part included questions regarding the perceived burden resulting from caregiving; the third part asked questions on caregivers’ coping resources; the fourth section focused on caregivers’ emotional experiences related to providing care for a child with a RD; the fifth part included questions regarding parents’ perceptions of their caregiving role, and the last part was related to their perceived quality of life (Supplementary material).

The religiosity of caregivers was assessed using two items: one regarding declared religious affiliation and the other concerning the significance of religion in their lives. Based on their responses, participants were categorized into two groups: the religious, including those caregivers who identified as members of a particular religious congregation and who declared that religion was important in their lives and influenced their decisions and choices, and the ambivalent/nonreligious which included caregivers who identified as nonbelievers for whom religion had little or no significance. However, religiosity is not a binary variable but a complex and continuous phenomenon consisting of a variety of different beliefs and practices that may vary considerably in terms of frequency and degree of importance in one’s life (Głaz, 2021). All questions were formulated in simple language and scored using a 5-point Likert scale ranging from 1 (strong dissatisfaction or disagreement) to 5 (high satisfaction or agreement).

### Data Collection

The research co-ordinator firstly contacted various RD foundations, patient associations, and organizations were contacted to determine whether they were interested in taking part in the survey. Once permission was granted, a letter of invitation and the online version of the questionnaire were posted on their web pages or Facebook pages, and all affiliated parents of children with a RD who met the inclusion criteria were invited to participate in the survey. Once the caregivers provided written informed consent to participate, the survey was completed via electronic devices (for example, computers, tablets, or smartphones), which took approximately 15–20 min. Two follow-up messages were sent in January and March.

### Data Analysis

The collected information was reviewed to ensure accuracy, consistency, and comprehensiveness before being processed and imported into JASP (Version 0.18.0) and PQStat (Version 1.8.4) for rigorous statistical assessment. The results are presented through descriptive statistical analyses, while the relationships between variables are assessed by calculating odds ratios (OR) and 95% confidence intervals (CI). For the caregivers’ mean age and standard deviation (SD), 95% CI were computed using 10,000 bootstrap replicates. While more than one caregiver per household could complete the survey, no dyads participated in the study.

## Results

A total of 925 caregivers completed the survey, of which 867 were women, mainly mothers (93.7%) and 58 men (6.3%), and all were of Polish origin (mean age: 37.36 years, range: 19–72, SD = 6.07) (Table 1). All caregivers provided care for over 1002 children with RDs experiencing a variety of different diseases (Table 2) with 92.1% providing care for one child with a RD and 7.9% cared for two or more children with RDs. Most caregivers (67%) had completed a university degree, of which, 6.8% received medical degrees and 54.6% of caregivers reported being unemployed, including 52.5% due to caregiving responsibilities. Overall, 35.8% of participants lived in towns with up to 10,000 inhabitants, followed by large cities with over 500,000 inhabitants (19.8%) and cities with 10,000 to 50,000 inhabitants (18.4%). Most caregivers (86.8%) were in a relationship with a parent of a child with a RD (77.4% in marriage and 9.4% in a partnership). While most caregivers identified themselves as Roman Catholics (78.7%), 11.8% declared themselves either agnostic or atheist (2.9% and 8.9% respectively) and 61.7% declared that religion had little if any significance in their lives.

**Table 1:** Caregivers’ demographic characteristics

Characteristics	Caregivers N (%)
*Caregiver’s sex*	
Female	867 (93.7)
Male	58 (6.3)
*Caregiver’s age*	
Range	19–72
Median	37
IQR (1–3)	33–41
Mean (95%CI)	37.36 (36.96–37.77)
SD (95%CI)	6.07 (5.72–4.44)
*Number of children with RDs*	
1	852 (92.1)
2	69 (7.5)
3 or more	4 (0.4)
*Education*	
Primary school	7 (0.8)
Vocational school	41 (4.4)
High school	257 (27.8)
University	557 (60.2)
Medical university	63 (6.8)
*Professional activity*	
Employed part-time	67 (7.2)
Employed full-time	345 (37.3)
Unemployed	19 (2.1)
Unemployed due to childcare	486 (52.5)
Pension	8 (0.9)
*Place of residence*	
Up to 10,000 inhabitants	331 (35.8)
10–50,000 inhabitants	170 (18.4)
51–100,000 inhabitants	110 (11.9)
101–500,000 inhabitants	131 (14.2)
Above 500,000 inhabitants	183 (19.8)
*Marital status*	
Single	22 (2.4)
A partnership with parent of a child with a RD	87 (9.4)
A partnership with person who is not parent of a child with a RD	17 (1.8)
Married to parent of a child with a RD	716 (77.4)
Married to person who is not parent of a child with a RD	35 (3.8)
Widowed	5 (0.5)
Divorced	43 (4.7)
*Confession*	
Roman Catholic	728 (78.7)
Other Christian	21 (2.3)
Non-Christian	0 (0)
Believer but not belonging to any denomination	67 (7.2)
Agnostic	27 (2.9)
Atheist	82 (8.9)
*Significance of religion in personal life*	
Significant	105 (11.4)
Rather big	249 (26.9)
Little	383 (41.4)
None	188 (20.3)

**Table 2:** List of rare diseases

Name of disease	ORPHA code	Number of caregivers	Disease prevalence
Phenylketonuria, mild hyperphenylalaninemia	79,254	176	1 in 15 000
Dravet syndrome	33,069	72	1 in 30 000
22q11.2 deletion syndrome	567	44	1 in 4 500–10 000
Duchenne muscular dystrophy	98,896	38	1 in 3 500–9 300
Williams syndrome	904	33	1 in 7 500
Cystic fibrosis	586	24	1 in 5 000–9 000
Turner syndrome	881	20	1 in 2 000
Tuberous sclerosis	805	20	1 in 10 000
Ehlers-Danlos syndrome	98,249	18	1 in 3 100–4 500*
Sotos syndrome	821	18	1 in 14 000
Marfan syndrome	558	17	1 in 5 000
Hemophilia	448	16	1 in 12 000
Achromatopsia	49,382	12	1 in 30 000–50 000
Glycogen storage disease	79,201	12	1 in 20 000–43 000*
Long-chain 3-hydroxyacyl-CoA dehydrogenase deficiency	5	12	1 in 120 000–250 000
Kabuki syndrome	2322	10	1 in 32 000
Medium-chain acyl-CoA dehydrogenase deficiency	42	9	1 in 14 600
Smith-Magenis syndrome	819	9	1 in 15 000–25 000
Joubert syndrome	475	8	1 in 100 000
Wilson disease	905	8	1 in 30 000–110 000
Noonan syndrome	648	7	1 in 2 500
Rett syndrome	778	7	1 in 11 100–100 000
Wolf-Hirschhorn syndrome	280	7	1 in 20 000–50 000
Coffin-Siris syndrome	1465	6	1 in 1 000 000
Cri du chat syndrome	281	6	1 in 15 000–45 000
Loeys-Dietz syndrome	60,030	6	1 in 100 000*
Mucopolysaccharidosis	79,213	6	1 in 100 000
other		304	–

Sources: www.orpha.net, * www.ncbi.nlm.nih.gov, www.ehlers-danlos.com, www.radiopaedia.org

Most (97.8%) respondents displayed strong emotional engagement in their caregiving role, indicating a strong emotional connection to (Table 3). This association is statistically significant (*p* < 0.05), with an odds ratio (OR) of 3.590 and a 95% confidence interval (CI) between 1.044 and 12.340. Furthermore, most caregivers reported experiencing a variety of challenges related to their children’s condition, including physical (68%), lack of time for themselves (70.7%), and work restrictions (57.9%). Over half the participants were also burdened by their child’s mental, cognitive, and emotional challenges (53.9%), and complained about the healthcare system for children with RDs (50.7%), family finances, and lack of emotional support (50.3% apiece).

**Table 3:** Association between caregivers’ religiosity and perceived burden

Characteristics	Number of respondents who responded affirmatively (Percentage of the total)	OR religious vs. nonreligious	95%CI	*p*
Emotional engagement in caregiving role	905 (97.8%)	3.590	1.044–12.340	< 0.05
*Challenges related to caregiving for a child with a RD*				
The child’s physical condition	629 (68%)	0.964	0.726–1.281	ns
The child’s mental/cognitive/emotional condition	499 (53.9%)	0.912	0.700–1.191	ns
The child’s behaviours resulting from RD	426 (46.1%)	0.963	0.738–1.257	ns
Contacts with the healthcare system	469 (50.7%)	0.810	0.621–1.056	ns
Problems with daily activities	331 (35.8%)	0.911	0.690–1.202	ns
Family finances	465 (50.3%)	0.880	0.675–1.148	ns
Lack of emotional support	467 (50.5%)	0.917	0.704–1.196	ns
Home maintenance	417 (45.1%)	0.936	0.717–1.222	ns
Raising healthy children	140 (15.1%)	0.945	0.652–1.371	ns
Lack of time for myself	654 (70.7%)	0.798	0.598–1.066	ns
Work-related difficulties	332 (35.9%)	0.868	0.658–1.147	ns
Work restrictions	536 (57.9%)	1.017	0.777–1.330	ns

ns: not significant

The immediate family was the main source of social support for both religious and non-religious groups, with the former indicating it more frequently (Table 4). Religious caregivers were also more likely to indicate other sources of social support, including the rare disease association, an online support community, nearby residents, the family physician, religion, and a priest.

**Table 4:** Association between caregivers’ religiosity and social support resources

Caregivers’ social support system	Number of respondents who responded affirmatively (Percentage of the total)	OR religious vs. nonreligious	95%CI	*p*
Immediate family	601 (65%)	1.640	1.232–2.182	< 0.001
Extended family and friends	231 (25%)	1.173	0.866–1.590	ns
Rare diseases association/foundation	135 (14.6%)	1.610	1.115–2,540	0.01
Mental health professional	80 (8.6%)	0.627	0.379–1.037	ns
Local support group	33 (3.6%)	1.543	0.769–3.094	ns
Online support community	291 (31.5%)	1.418	1.068–1.881	< 0.05
Nearby residents	54 (5.8%)	1.800	1.038–3.124	< 0.05
Family physician	215 (23.2%)	1.379	1.012–1.879	< 0.05
Other medical practitioners	102 (11%)	1.502	0.993–2.271	ns
A priest	34 (3.7%)	28.273	6.732–118.747	< 0.0001
Someone else	75 (8.1%)	1.951	1.214–3.136	< 0.01
Religion / spirituality	138 (14.9%)	47.331	21.784–102.837	< 0.001
Personal interests and hobbies	185 (20%)	1.127	0.811–1.565	ns

ns: not significant

Regardless of their religiosity, the most frequently reported emotions experienced by all caregivers were fear about the progression of their children’s disease (69.1%), helplessness (64.8%), and anxiety and fear (64.4%) (Table 5). Many parents, however, also reported experiencing impatience and irritation (47.1%), emotional lability (47%), sadness and depression (41.6%), or nervousness and impulsivity (41.5%). Also, 45.4% felt that their needs were unimportant to others, with non-religious caregivers reporting experiencing anger (*p* < 0.01) and impatience and irritation (*p* < 0.05) more often.

**Table 5:** Association between caregivers’ religiosity and experienced emotions

Do you experience any of these emotions resulting from caring over a child with a RD?	Number of respondents who responded affirmatively (Percentage of the total)	OR religious vs. nonreligious	95%CI	*p*
Emotional lability	435 (47%)	0.872	0.668–1.137	ns
Emotional control problem	353 (38.2%)	0.854	0.649–1.124	ns
Impatience/irritation	436 (47.1%)	0.760	0.582–0.993	< 0.05
Nervousness/impulsivity	384 (41.5%)	0.828	0.632–1.085	ns
Anger	245 (26.5%)	0.650	0.477–0.887	< 0.01
Anxiety/fear	596 (64.4%)	1.018	0.772–1.344	ns
Helplessness	599 (64.8%)	0.975	0.739–1.287	ns
Sadness/depression	385 (41.6%)	0.822	0.627–1.078	ns
Lack of self-confidence	300 (32.4%)	1.069	0.806–1.418	ns
My own needs are not important to others	420 (45.4%)	0.883	0.676–1.153	ns
Hopelessness	328 (35.5%)	0.761	0.574–1.007	ns
Loss of meaning in what you do	224 (24.2%)	0.761	0.555–1.044	ns
Feeling guilty	286 (30.9%)	1.034	0.776–1.377	ns
Shame	87 (9.4%)	0.789	0.495–1.259	ns
Loneliness	359 (38.8%)	0.937	0.713–1.230	ns
A desire to retreat from the environment	274 (29.6%)	0.918	0.686–1.229	ns
Low self-esteem	261 (28.2%)	0.937	0.697–1.259	ns
Fear over the progression of child’s disease	639 (69.1%)	1.227	0.918–1.640	ns
Anticipatory loss	366 (39.6%)	1.038	0.791–1.360	ns

ns: not significant

Caregivers’ religiosity was also associated with their perceptions of the role of the caregiver (Table 6). Most participants declared that caregiving for a child with a RD is mentally challenging (62.7%) and physically exhausting (58.5%), and felt that nobody understood what they were going through (55.5%), complaining that they had to give up their personal plans, passions, and hobbies (53.9%). Nonetheless, 50.2% of the respondents declared that caregiving made them stronger as a person.

**Table 6:** Association between caregivers’ religiosity and perception of caregiving role

How do you perceive your caregiving role?	Number of respondents who responded affirmatively (Percentage of the total)	OR religious vs. nonreligious	95%CI	*p*
Caregiving for my child with a RD is a source of personal satisfaction	305 (33%)	1.138	0.859–1.507	ns
Caregiving made me stronger as a person	464 (50.2%)	1.256	0.963–1.638	ns
My child’s disease had positive impact on my life	199 (21.5%)	1.647	1.200–2.261	< 0.01
I consider it my moral duty to care for my child with a RD	854 (92.3%)	1.321	0.789–2.212	ns
Caregiving for my child with a RD is physical exhausting	541 (58.5%)	0.999	0.763–1.308	ns
Caregiving for my child with a RD is mentally challenging	580 (62.7%)	0.808	0.615–1.061	ns
Caring for my child with a RD is frustrating	200 (21.6%)	0.791	0.569–1.098	ns
I feel lack of emotional connection with my child with a RD	224 (24.2%)	0.761	0.555–1.044	ns
I feel uncomfortable when people are in the presence of my child with a RD	122 (13.2%)	0.583	0.383–0.888	0.01
I feel uncomfortable when dealing with the hygiene of my child with a RD	84 (9.1%)	0.886	0.555–1.415	ns
Caregiving for my child with a RD is a source of stress	263 (28.4%)	0.860	0.639–1.157	ns
I am not coping well with the stress	318 (34.4%)	0.820	0.619–1.087	ns
I experience care overload	358 (38.7%)	0.676	0.513–0.892	< 0.01
Caregiving role is beyond my abilities	190 (20.5%)	1.096	0.791–1.519	ns
My own needs are not important to others	420 (45.4%)	0.883	0.676–1.153	ns
Nobody understands what I am going through	513 (55.5%)	0.842	0.645–1.099	ns
My entire life is subordinated to the role of caregiver	528 (57.1%)	0.741	0.567–0.969	< 0.05
I experience conflict between my own needs and those of my a child’s with a RD	271 (29.3%)	0.805	0.599–1.081	ns
Caregiving makes it hard for me to fulfil other roles, i.e. parent/spouse/employee	332 (35.9%)	0.723	0.546–0.959	< 0.05
Caregiving for my child with a RD is a source of social exclusion	132 (14.3%)	0.779	0.528–1.149	ns
Because of caregiving I had to abandon my plans, passions and hobbies	499 (53.9%)	0.930	0.713–1.213	ns

ns: not significant

The most frequently reported perception of the role of caregiving was considering it a moral duty to care for a child with a RD (92.3%), and this perception was consistent among both religious and non-religious groups (*p* < 0.01). Non-religious caregivers reported feeling that their entire life was subordinated to the role of caregiver more often (*p* < 0.05), as well as experiencing care overload, and declared that caregiving made it hard to fulfill other roles (*p* < 0.05). They also felt more uncomfortable when other people were in the presence of their child with a RD (*p* < 0.01), whereas religious caregivers reported that their child’s disease had a positive impact on their life (*p* < 0.01).

All caregivers’ reported the impact of caregiving on their perceived quality of life and several risks resulting from caregiving (Table 7). The most frequently mentioned problem was tiredness and exhaustion (78.4%) with more than half of respondents feeling insecure (58.9%), preoccupied with their financial situation (56.9%), physical health (56.1%) and reported a decreased quality of life (54.4%).

**Table 7:** Association between caregivers’ religiosity and perceived quality of life

	Number of respondents who responded affirmatively (Percentage of the total)	OR religious vs. nonreligious	95%CI	*p*
*Risks and problems resulting from caregiving for a child with a RD*				
Decreased appetite	164 (17.7%)	1.141	0.809–1.609	ns
Weight loss / weight gain	310 (33.5%)	0.872	0.657–1.157	ns
Tiredness / exhaustion	725 (78.4%)	1.071	0.775–1.481	ns
Sleeplessness / sleep problems	393 (42.5%)	0.870	0.665–1.139	ns
Decline in health	389 (42.1%)	1.081	0.826–1.413	ns
Decline in mental health	434 (46.9%)	0.730	0.558–0.953	< 0.05
Disturbance of intimacy in a relationship	396 (42.8%)	0.885	0.676–1.157	ns
Substance abuse	92 (9.9%)	0.607	0.377–0.975	< 0.05
*Perceived quality of life*				
Life situation	654 (70.7%)	1.722	1.271–2.333	< 0.001
Life satisfaction	528 (57.1%)	1.767	1.344–2.323	< 0.0001
Physical health	519 (56.1%)	1.026	0.785–1.340	ns
Personal well-being	448 (48.4%)	1.458	1.117–1.903	< 0.01
Sense of security	545 (58.9%)	1.291	0.984–1.694	ns
Financial situation	526 (56.9%)	1.293	0.988–1.693	ns
Family relationships	736 (79.6%)	1.776	1.252–2.519	0.001
Interactions with friends	403 (43.6%)	1.199	0.918–1.566	ns
Quality of sleep	405 (43.8%)	1.321	1.012–1.725	< 0.05
Sense of satisfaction, achievement and personal growth	246 (26.6%)	1.094	0.812–1.475	ns
Decreased quality of life	503 (54.4%)	0.809	0.620–1.056	ns
Personal satisfaction	468 (50.6%)	1.524	1.167–1.990	< 0.01
Personal happiness	539 (58.3%)	1.573	1.196–2.067	0.001

ns: not significant

However, while the non-religious caregivers reported a decline in mental health and an increase in substance abuse, they also reported a poorer quality of sleep (*p* < 0.05). In terms of various aspects related to the quality of life, family relationships were assessed most positively, whereas religious caregivers tended to assess family relationships more positively (*p* < 0.01). They also perceived the following aspects of their life more positively: personal satisfaction (*p* < 0.01), personal happiness (*p* = 0.001), personal well-being (*p* < 0.01) and life satisfaction (*p* < 0.0001).

## Discussion

Since most sufferers of rare diseases are children, they are cared for at home by their families (Ministerstwo Zdrowia, 2021). Consequently, caregivers of children with RDs are *on-call* 24 h a day, seven days a week, and face numerous challenges related to their children’s health problems, psychiatric conditions, mood swings and behaviors (Scallan et al., 2011; Reilly et al., 2015; Bonner et al., 2017; Sandilands et al., 2022; von der Lippe et al., 2022; Bösch et al., 2022; Atkins & Padgett, 2024). Previous research demonstrated serious physical, mental, emotional, psychological, social, and economic costs of caregiving, and that parents of children with RDs experienced high levels of burden and had a significant number of unmet needs (Pelentsov et al., 2016a, 2016b; Mcmullan, Lohfeld & 2022; Kowal et al., 2022; Witt et al., 2023). Similarly, while Polish caregivers of children with RDs enrolled in this study reported a lack of time for themselves, being burdened with their child’s physical, mental, cognitive, and emotional state, many were also concerned about the deterioration of their physical and mental health, family finances and lack of emotional support (Bonner et al., 2017; Boettcher et al., 2020; Boettcher et al., 2021; Sandilands et al., 2022; von der Lippe et al., 2022; Bösch et al., 2022; Atkins & Padgett, 2024).

This was unsurprising since numerous studies have reported that caregivers complain about bodily pain, lack of energy for daily activities, and a decline in physical and mental health (Domaradzki & Walkowiak, 2023a; Kowal et al., 2022; Michalíka, 2014; Sevin et al., 2022; Valcárcel-Nazco et al., 2022; Xu et al., 2021). Some also report decreased engagement in preventative behaviors, poor diet, and increased risk of substance abuse, including smoking, drinking alcohol, and use of prescribed medications (Mcmullan et al., 2022; Rihm et al., 2022; Sandilands et al., 2022). Caregivers of children with RDs have poor patterns of sleep related to constant thinking about and preoccupation with the progression of their child’s disease and anticipated death (Dias et al., 2023; Domaradzki & Walkowiak, 2023a).

The present study also revealed that the caregivers of children with RDs were profoundly engaged in caregiving and felt a deep emotional connection to their children, experiencing a variety of distressing emotions. This is in line with previous studies, which demonstrated that because many caregivers lack both informal and formal support, and caring over a child with a RD is characterized by the number, severity, and longitude of symptoms, duration, and multitude of caregiving tasks, caregivers have cumulative demands of numerous roles which increases their feeling of being alone (Currie & Szabo, 2019a, 2019b). Since caring for a child with a RD is more demanding and stressful than caring for children with other disabilities, caregivers also experience role captivity and role overload, becoming physically and emotionally overwhelmed (Kowal et al., 2022; Pelentsov et al., 2015, 2016a, 2016b). Consequently, there is a strong correlation between caring for a child with a RD and psychological morbidity, including depression and anxiety, which are often characterized by feelings of fear, sadness, anger, loneliness, fatigue, and sleep problems (Boettcher et al., 2020; Domaradzki & Walkowiak, 2023b; Lai et al., 2023; Lai et al., 2023; Lanfranchi et al., 2012; Lazow et al., 2019; Moraleda Sepúlveda & López Resa, 2021; Picci et al., 2015; Rihm et al., 2022; Williams et al., 2009), especially since they have little time for respite and are often compelled to abandon their leisure activities, give up vacations or hobbies (Cardinali et al., 2019; Mcmullan et al., 2022).

Most studies on caregivers of children with RDs focus on parental stress and the caregiving burden, and providing care for an individual with RD is framed as having mainly negative outcomes in terms of increased psychological distress, higher burden, and decreased quality of life. Only recently has caregiving been understood in terms of the “stress-appraisal-coping” paradigm, which stresses that since one’s perception of caregiving depends both on the meaning a person attaches to the caregiving, the stressor and coping resources available to deal with these stressors (Lazarus & Folkman, 1984). Consequently, it is suggested that the transaction between caregiving, personal resources, and the environment may result in a caregiving appraisal that may be positive, negative, or neutral.

Thus, even though this study shows that most family caregivers reported that the challenges related to parenting a child with a RD had many negative psychosocial consequences and lower quality of life in all members of the RD family, it also confirms that some parents reported more positive caregiving experiences (Somanadhan & Larkin, 2016; Vitale, 2016; von der Lippe et al., 2022; Atkins & Padgett, 2024). Some parents emphasized the positive dimension of caregiving for a child with a RD, suggesting that it was a source of personal satisfaction, made them stronger as a person, or had a positive impact on their lives. Similarly, a previous study demonstrated that while caregivers of patients with Pompe disease reported experiencing many problems with daily activities and physical and/or mental problems, they also stressed that caregiving was a source of personal fulfillment. Additionally, many declared that they would become unhappy if someone else were to provide care for their relatives (Kanters et al., 2013). Also, parents of children with Willimas syndrome emphasized that although caregiving is associated with many daily challenges it was a rewarding experience and had a positive impact on their families, as it brought all family members closer, helped them to appreciate life, enabled personal growth and resulted in friendship with other parents (Reilly et al., 2015; Scallan et al., 2011). Finally, parents of children with neuronal ceroid lipofuscinosis 3 (CLN3) disease reported positive implications of the disease such as an increased sense of empathy and responsibility in the healthy sibling (Krantz et al., 2022).

This study also showed that although the family is the main source of support for caregivers of children with RDs, raising such a child may damage family structure and social contact with other family members and friends. This is in line with previous studies that reported caregiving damages parents’ social interactions, they often feel lost, emotionally and socially isolated, and lonely, and these feelings increase as their child’s disease progresses (Nolan et al., 2006; Michalíka, 2014; Currie & Szabo, 2020; von der Lippe et al., 2022; Dias et al., 2023; Hatirnaz Ng et al., 2023). The child’s disease also changes family dynamics, is a predictor of conflict between parents, and may lead to a lack of intimacy and emotional closeness (Domaradzki & Walkowiak, 2023a). It also provokes family disharmony and contributes to increased tensions with other family members over the way caregiving is provided and, the lack of social and financial support (Cardinali et al., 2019; Mcmullan et al., 2022). Caregivers’ of children with RDs perception of the quality of their social life is significantly poorer than those of other caregivers (Boettcher et al., 2021; Reilly et al., 2015).

Finally, as many caregivers enrolled in this study were preoccupied with their financial situation and complained about work restrictions resulting from caregiving, this study is in line with previous studies that reported harmful consequences of raising a child with a RD on family finances and professional life, including job loss (Bonner et al., 2017; Walkowiak et al., 2023). Indeed, many studies have documented the multi-billion dollar burden of RD which includes both direct and indirect medical costs, the caregiver’s excess health costs such as services provided outside the medical care system, and direct and indirect social costs, that is, the value of caregiving time, the caregiver’s loss of employment and income, work-related difficulties, including missing work, being tired at work, missing new job opportunities or chances of promotion (Pelentsov et al., 2016a, 2016b; Linertová et al., 2017; Yang et al., 2022). Due to their caregiving responsibilities, many parents have to limit or give up their work, reporting a significant deterioration in their economic status (Hatirnaz Ng et al., 2023; Michalíka, 2014). This is important since previous research conducted among family caregivers of children with phenylketonuria demonstrated that while parents experience job restrictions or loss of jobs, there is a reassuring link between subjective happiness, financial situation, and professional activity among female caregivers. It was also shown that this relationship goes far beyond the income as professional activity has a therapeutic value for caregivers (Walkowiak et al., 2023).

Most importantly, this research shows that as RD progresses and caregivers experience prolonged anxiety, depression, and anticipated loss, religion may serve as a positive adaptive strategy for coping with the stress and caregiving burden (Boettcher et al., 2021; Leidl et al., 2023; Pearce, 2005; Pearce et al., 2016; Picci et al., 2015; Sandilands et al., 2022). Although over the years many Poles have turned away from the Catholic Church but not from religious beliefs, Catholic religiosity has shaped Polish culture and is highly institutionalized, both at the public and private level, thus is an effective coping resource for family caregivers.

At the same time, although religion and spirituality are often used interchangeably they should be distinguished. While religiosity involves engaging in beliefs and practices specific to organized religious tradition, for example, the Roman Catholic Church, and usually emphasizes the importance of belonging, community, shared identity and beliefs, and collective worship, spirituality is more complex and is often defined more a personal and subjective journey which involves a personal quest for meaning in life and a direct relationship with the divine. Thus, it does not need a community of followers, nor does it adhere to established rules, but instead it is more fluid and encourages the individual to explore one’s inner self and establish a personal and profound connection with the sacred or transcendent, whether God, supreme-being, higher power or the spiritual world (Hill & Pargament, 2003; Koenig et al., 2012). Similarly, while religious coping refers to the use of faith, particularly turning to God, religious beliefs or practices that aim to seek comfort, support, and guidance from a divine and to cope with difficult life situations usually within the domain of organized religion (Pargament et al., 2000), spiritual coping refers to a more informal path through one’s spirituality that allows individuals to find meaning and purpose in life. Thus, it requires neither doctrine nor organizational background and refers to such concepts as autonomy, dignity, meaningful activities, and social relationships (Leidl et al., 2023).

Indeed, while some studies have reported a deleterious effect of religiosity in coping and dealing with stressful situations (Pargament et al., 2000), showing that some religious caregivers of children with life-limiting or life-threatening disease might interpret their child’s disease as a punishment from God for their mistakes or sins, or as a possession by the devil or evil spirits (Grossoehme et al., 2010b; Daher-Nashif et al., 2020; Malcolm et al., 2021) or may experience a sense of divine or spiritual abandonment (Nicholas et al., 2017; Chong, Khalid & Abdullah, 2022), this study shows that religious caregivers reported less of a feeling of burden and stressed that their child’s disease had a helpful impact on their life. This is in line with the observation made by Grossoehme et al. (2010b) who reported that while parents of children with cystic fibrosis adopted sixteen religious coping styles, positive religious coping styles (pleading, collaboration, benevolent religious reappraisals, and seeking spiritual support) were more frequent than negative styles. Similarly, although both parents of children with Prader-Willi syndrome and epidermolysis bullosa struggled to care for themselves and their families, they often stressed that religion, spirituality, and meditation were a source of support and assistance and important coping resource (Vitale, 2016; Wu et al., 2020).

Another important finding is that religion may have a positive influence on the caregiving experience because it increases the caregivers’ ability to give meaning and process distressing events in life, supports parents, and helps them to accept difficult life situations. Purcell and colleagues, for example, demonstrated that parents of children diagnosed with neuroendocrine hyperplasia in infancy used their faith to cope and give meaning to their child’s disease and believed that it occurred for a specific purpose (Purcell et al., 2015). Similarly, parents of children with cystic fibrosis coped with their children’s disease by giving religious meaning to parenting as a vocation according to God’s plan (Grossoehme et al., 2012). In another study, religion helped parents to experience their disabled children not as a loss but as a gain. It also positively influenced closeness with a child and parental care (Durà-Vilà et al., 2010).

Another important finding is that although both parents who declared themselves as religious and non-religious felt burdened by the role of caregiving, it was the former who declared having had more support available, relying not only on family, neighbors, online support community, RD association, and a family physician but stressed support from religion and priests. This is of particular importance since much research demonstrated that especially during the first stages of disease, caregivers of children with RDs receive insufficient information about diagnosis, disease trajectory, its treatment or available formal services and constantly struggle with the diagnostic and therapeutic odyssey (Black, Martineau & Manacorda, 2015; Carmichael et al., 2015; Bauskis et al., 2022; Llubes-Arrià et al., 2022; Teutsch et al., 2023). While securing proper medical help for their children is the main unmet need of many caregivers, they also face problems in communication and complain of the lack of emotional support and support from healthcare professionals and psychological counseling (Currie & Szabo, 2019a, 2019b; Domaradzki, 2016; Teutsch et al., 2023; Witt et al., 2023).

Since parents of children with RDs often experience isolation, exclusion, and marginalization within the family and public domains, including medical and social systems and healthcare and government disability support providers (Currie & Szabo, 2020; Kolset, 2016), they have limited access to other coping resources, and for that reason, they may benefit from religion, which offers moral support. Since the diagnostic and therapeutic journey in RD is a time of uncertainty and helplessness for many caregivers, religion becomes a source of strength during the difficult process of diagnosis (Durà-Vilà et al., 2010; Purcell et al., 2015; Vitale, 2016; Wu et al., 2020). Dogan (2016), for example, showed that religious beliefs are helpful to parents in making sense of their children’s disability and coping with difficulties by providing explanations for the meaning of life. They also help them gain confidence. Grossoehme et al. (2010a) reported that religious parents of children with cystic fibrosis who imagined God as active, benevolent, and interventionist felt supported by God, and religion helped them find hope, gave some sense of control and motivation to care (Grossoehme et al., 2010a). Similarly, Nematollahi et al. (2021) showed that while spiritual beliefs shaped Iranian parents' experience of their children with phenylketonuria, as they felt being closer to God, did more worship and relied more on God, it was also an important part of the caring process. Moreover, most parents declared that their child’s disease strengthened their faith.

This study also shows that religion was associated with a more positive caregiving experience and was a source of comfort, strength, and guidance and may be an important contributor to caregiver emotional adjustment (Durà-Vilà et al., 2010; Pargament et al., 2000). While both religious and nonreligious caregivers reported experiencing a variety of distressing emotions resulting from caregiving, the latter reported anger, impatience, and irritation more often. It therefore supports others’ observations that religious coping strategies increase caregivers’ level of satisfaction and are associated with encouraging emotions, improved mood and mental health (Pearce et al., 2016). Hebert and colleagues, for example, found that religious beliefs and practices (prayer and meditation), and attendance at religious services in particular were positively associated with better mental health in caregivers of individuals with dementia (Hebert et al., 2007).

Other studies showed that religious coping is associated with improved mood, a more positive perception of the role of caregiving and serves as a protective factor against depression in caregivers of persons with serious mental illness, Alzheimer’s disease, and dementia (Murray-Swank et al., 2006; Nightingale, 2003; Yoon et al., 2018). Also, Liu et al. (2021) reported that while religious coping was the most common method used by parents of infants with congenital heart disease who experienced emotional disorders, positive religious thoughts, and coping methods were associated with a decreased likelihood of experiencing depression, and negative religious thoughts and religious coping increased the risk of depression among the parents. Similarly, while Engel et al. reported that parents of children with a life-limiting or life-threatening disease stressed the spiritual dimension of parenting, having a diseased child was both a source of spirituality and the cause of spiritual concerns (Engel et al., 2023).

Finally, this research confirms the association between caregivers’ religiosity and their perceived quality of life. Although parents from both groups described caregiving for RD children as physically, emotionally, and mentally challenging, reporting tiredness and exhaustion, religious caregivers perceived more aspects of their lives positively, while non-religious caregivers experienced role captivity and role overload more often. As religious caregivers declared that their children’s disease had a positive impact on their lives, this study also supports the observation that caregivers with higher levels of personal religiosity report higher levels of mastery, and self-esteem and associate a light burden and higher satisfaction with the role of caregiving (Xavier & Esperandio, 2023). Other research suggests that because religion helps caregivers develop more positive or hopeful expectations, religious coping may ameliorate hopelessness and is also positively associated with caregivers’ increased level of contentment and general well-being (Fider et al., 2019; Leidl et al., 2023; Pearce, 2005; Pearce et al., 2016; Vigna et al., 2020). Calvo et al., for example, demonstrated that religiousness was positively associated with amyotrophic lateral sclerosis caregivers’ quality of life and satisfaction with life (Calvo et al., 2011). Parents of children with spinal muscular atrophy used religion as a coping resource to deal with helplessness, loss of hope, suffering, and anticipatory grief resulting from their child’s rare condition (Yang et al., 2016). Also, spirituality, religious beliefs, and practices, including yoga or mindfulness, helped caregivers revisit life’s challenges, accept trials and tribulations, and find the strength to cope with their child’s disease (Purcell et al., 2015; Vitale, 2016; Wu et al., 2020).

Another study on Italian caregivers of persons with epidermolysis bullosa found that one of the risk factors associated with the caregiving burden was not being religiously affiliated. The caregivers who identified as religious declared that religious communities allowed them to create new social networks, where they could meet new friends who would provide material assistance, understand their situation, and recognize their child as special (Tabolli et al., 2010). Another study on Iranian mothers of children with recurrent leukemia found that religious coping was significantly associated with their quality of life and that religious support improved their quality of life (Khanjari et al., 2018). Similarly, a recent study by Leidl et al. (2023) showed that as caregivers of persons with Huntington’s disease progressively lose their loved ones, they experience prolonged distress, existential angst, grief, and other forms of spiritual suffering, frequently having maladaptive coping strategies characterized by dysfunctional escape schemes.

## Study Limitations

Although to the best of our knowledge, this is one of the few studies to assess the association between religiosity and the caregiving experiences of parents with a child with a rare disease in Poland, it also has its limitations. Most importantly, although the response rate was reasonably high, with 925 individual responses, the estimated number of RD children in the country is much higher, thus these results represent only the opinions of those parents who took part in the survey and cannot be extrapolated to the entire population of Polish caregivers of children with RDs. However, it should be acknowledged that although one of the main objectives of the *National Plan for Rare Diseases* adopted by the Polish government is to create a Register of Rare Diseases, there remains no such register and for that reason, the exact number of children with RDs is unknown. Additionally, since RDs cover over 10,000 conditions differing significantly in terms of variety, complexity, and severity of symptoms, disease trajectory, and prognosis, it is difficult to compare the experiences of all caregivers who experience radically different situations regarding taking care of a sick child. Thus, since those different circumstances can also affect parents’ religiosity, future studies should be conducted on a more unified group of caregivers. Future research should also explore the impact of having a child with a rare condition on parents’ religiosity. However, it should be acknowledged that because parents’ experiences are often neglected by the Polish healthcare system, and there are no previous studies on the role of religiosity as a coping resource for managing the stress related to caregiving of RD child, we believe that allowing all RD parents, irrespective of their child’s condition, to share their experiences is also important. Secondly, because many caregivers were uninterested in participating in the study or did not want to reveal information regarding their circumstances and experiences, this survey reflects only the opinions of those parents who agreed to take part in the study and share their experiences. Thirdly, since most caregivers enrolled in this study identified themselves as Roman Catholics (78.7%) and Poland is a predominantly Catholic nation, the results cannot be generalized to other cultures and faith traditions. Fourthly, because we assessed the caregiving experiences of parents with children aged under 18, the results may not reflect the opinions of parent caregivers of adult patients, which may be different. Since RDs display a high variety and complexity of symptoms and they differ in prognosis and available treatment options, they result in different health and psycho-social challenges that may be difficult to compare. It is therefore recommended that a comparison between the experiences of the parents of RD children, adolescents, and adults be performed. Fifthly, since only 58 male caregivers responded and completed the survey, these results underrepresented male caregivers’ perspectives. Previous research, however, confirms that there is a gender gap in caregiving (Chiarotti et al., 2023; Chu et al., 2022). Sixthly, even though a pediatrician, a public health expert, and a medical sociologist assisted in the development of the questionnaire, it was not validated.

Although there are several scales by which to measure religiosity, it was assessed using self-developed questions regarding declared religious affiliation and the other concerning the significance of religion in one’s life. However, we did not ask specific questions regarding caregivers’ religious beliefs and attendance. Thus, while there is a possibility of measurement error and caution should be exercised when interpreting the results, future studies should therefore use well-validated instruments that quantify the religious stance, caregiving burden, and quality of life that would help to establish construct validity and more rigorous and robust assessment. Additionally, the participants were categorized into two groups, religious and ambivalent/non-religious, which may suggest that religiosity is a binary rather than a continuous variable.

Both caregivers’ descriptions of their children’s symptoms and experiences were also self-assessed and self-reported, there is a risk of a misunderstanding as they may not reflect well-defined medical categories. The study concerning the burden of caregiving is based on what the caregivers report and, even though opinion data are useful, they represent solely subjective outcomes. At the same time, to further explore the experiences, challenges, and needs of caregivers of children with RDs, a more in-depth qualitative research project using methods such as an in-depth interview or focus groups should be conducted. Finally, since this research was designed as a computer-assisted online survey, there is potential recruitment bias as some caregivers do not use social media platforms, are not members of online support groups, associations, or organizations that helped in the recruitment of the participants, or do not feel comfortable using electronic devices and did not want to share their experiences.

Despite these limitations, this study also had several strengths. Most importantly, to the best of our knowledge, it was the first study of the experience of Polish family caregivers of children with rare diseases. This research shows that although caregivers are also impacted by the disease, they do not receive services or attention, therefore it sets a baseline for future research opportunities. Identifying parents of children with RDs as invisible patients may help identify actions required from the government, and medical and social institutions to alleviate the burden of caring for a child with a RD and improve caregivers’ quality of life. Finally, as acknowledged by many participants, since this study gave voice to parents and allowed them to share their perspectives, it had a therapeutic value.

## Conclusion

Caring for a child with a RD is extremely demanding and may lead to physical, mental, emotional, and behavioral problems in parents. Although caregivers adopt a variety of coping strategies, including psychological, cognitive, spiritual, and practical, the association of religiosity and the caregiving burden has been understudied in Polish studies. Nevertheless, parents’ religiosity is associated with a more positive caregiving experience, perceived quality of life, and experienced burden. While religious caregivers reported experiencing less distressing emotions and stressed the positive impact of their child’s disease on their life more often, non-religious caregivers experienced role captivity and role overload more frequently. This study therefore confirms that religion may serve as a source of strength, a positive adaptive strategy, and protect against mental health problems and caregiving burden. There is an urgent need for research on the impact of religious coping methods on caregivers of children with RDs, healthcare professionals should be aware of the importance of religious and spiritual care, and caregivers’ religiosity should be considered an integral part of a holistic approach (Carey & Mathisen, 2018; Puchalski et al., 2014). Religious and spiritual care for caregivers of children with RDs should be integrated into health and social policy systems.

## Supplementary Information

Below is the link to the electronic supplementary material.

Supplementary file1 (DOC 114 kb)

## Abbreviation

RD: Rare Disease
