# Supplementary material for: “In God We Trust”: An Exploratory Study of the Associations Between Religiosity and the Caregiving Experiences of Parents of Children with Rare Diseases in Poland
Source: J Relig Health. 2024 Aug 5;63(6):4079–109. doi: 10.1007/s10943-024-02095-4 (PMC11576783; doi:10.1007/s10943-024-02095-4)
Supplement: Supplementary file 1 — Supplementary file1 (DOC 114 kb) [file 10943_2024_2095_MOESM1_ESM.doc]

Supplementary material. Questionnaire

| *What makes caring for a child with rare disease challenging* | Never | Rarely | Sometimes | Often | Always |
| --- | --- | --- | --- | --- | --- |
| the child’s physical condition |  |  |  |  |  |
| the child’s mental/cognitive/emotional condition |  |  |  |  |  |
| the child’s behaviours resulting from RD |  |  |  |  |  |
| contacts with the healthcare system |  |  |  |  |  |
| problems with daily activities |  |  |  |  |  |
| family finances |  |  |  |  |  |
| lack of emotional support |  |  |  |  |  |
| home maintenance |  |  |  |  |  |
| raising healthy children |  |  |  |  |  |
| lack of time for myself |  |  |  |  |  |
| work-related difficulties |  |  |  |  |  |
| work restrictions |  |  |  |  |  |
| *How often can you relay on the following coping resources?* | Never | Rarely | Sometimes | Often | Always |
| immediate family |  |  |  |  |  |
| extended family and friends |  |  |  |  |  |
| rare diseases association/foundation |  |  |  |  |  |
| mental health professional |  |  |  |  |  |
| local support group |  |  |  |  |  |
| online support community |  |  |  |  |  |
| nearby residents |  |  |  |  |  |
| family physician |  |  |  |  |  |
| other medical practitioners |  |  |  |  |  |
| a priest |  |  |  |  |  |
| someone else |  |  |  |  |  |
| religion/spirituality |  |  |  |  |  |
| personal interests and hobbies |  |  |  |  |  |
| *Do you experience any of these emotions resulting from caring over a child with rare disease?* | Never | Rarely | Sometimes | Often | Always |
| emotional lability |  |  |  |  |  |
| emotional control problem |  |  |  |  |  |
| impatience/irritation |  |  |  |  |  |
| nervousness/impulsivity |  |  |  |  |  |
| anger |  |  |  |  |  |
| anxiety/fear |  |  |  |  |  |
| helplessness |  |  |  |  |  |
| sadness/depression |  |  |  |  |  |
| lack of self-confidence |  |  |  |  |  |
| my own needs are not important to others |  |  |  |  |  |
| hopelessness |  |  |  |  |  |
| loss of meaning in what you do |  |  |  |  |  |
| feeling guilty |  |  |  |  |  |
| shame |  |  |  |  |  |
| loneliness |  |  |  |  |  |
| a desire to retreat from the environment |  |  |  |  |  |
| low self-esteem |  |  |  |  |  |
| fear over the progression of child’s disease |  |  |  |  |  |
| anticipatory loss |  |  |  |  |  |
| *How do you perceive your caregiving role?* | Never | Rarely | Sometimes | Often | Always |
| caregiving for my child with rare disease is a source of personal satisfaction |  |  |  |  |  |
| caregiving made me stronger as a person |  |  |  |  |  |
| my child’s disease had positive impact on my life |  |  |  |  |  |
| I consider it my moral duty to care for my child with rare disease |  |  |  |  |  |
| caregiving for my child with rare disease is physical exhausting |  |  |  |  |  |
| caregiving for my child with rare disease is mentally challenging |  |  |  |  |  |
| caring for my child with rare disease is frustrating |  |  |  |  |  |
| I feel lack of emotional connection with my child with rare disease |  |  |  |  |  |
| I feel uncomfortable when people are in the presence of my child with rare disease |  |  |  |  |  |
| I feel uncomfortable when dealing with the hygiene of my child with rare disease |  |  |  |  |  |
| caregiving for my child with a RD is a source of stress |  |  |  |  |  |
| I am not coping well with the stress |  |  |  |  |  |
| I experience care overload |  |  |  |  |  |
| caregiving role is beyond my abilities |  |  |  |  |  |
| my own needs are not important to others |  |  |  |  |  |
| nobody understands what I am going through |  |  |  |  |  |
| my entire life is subordinated to the role of caregiver |  |  |  |  |  |
| I experience conflict between my own needs and those of my child’s with a RD |  |  |  |  |  |
| caregiving makes it hard for me to fulfil other roles, i.e. parent/spouse/employee |  |  |  |  |  |
| caregiving for my child with a RD is a source of social exclusion |  |  |  |  |  |
| because of caregiving I had to abandon my plans, passions and hobbies |  |  |  |  |  |
| *Risk and problems resulting from caregiving for a child with a RD* | Never | Rarely | Sometimes | Often | Always |
| decreased appetite |  |  |  |  |  |
| weight loss / weight gain |  |  |  |  |  |
| tiredness / exhaustion |  |  |  |  |  |
| sleeplessness / sleep problems |  |  |  |  |  |
| decline in health |  |  |  |  |  |
| decline in mental health |  |  |  |  |  |
| disturbance of intimacy in a relationship |  |  |  |  |  |
| substance abuse |  |  |  |  |  |
| *How do you perceive the following aspects of your quality of life?* | Very bad | Rather bad | Neither good nor bad/I do not know | Rather good | Very good |
| life situation |  |  |  |  |  |
| life satisfaction |  |  |  |  |  |
| physical health |  |  |  |  |  |
| personal well-being |  |  |  |  |  |
| sense of security |  |  |  |  |  |
| financial situation |  |  |  |  |  |
| family relationships |  |  |  |  |  |
| interactions with friends |  |  |  |  |  |
| quality of sleep |  |  |  |  |  |
| sense of satisfaction, achievement and personal growth |  |  |  |  |  |
| decreased quality of life |  |  |  |  |  |
| personal satisfaction |  |  |  |  |  |
| personal happiness |  |  |  |  |  |
